# 91. Gaps and Opportunities in Antimicrobial Stewardship Programs in Asia: A Survey of 10 Countries

**DOI:** 10.1093/ofid/ofab466.091

**Published:** 2021-12-04

**Authors:** Feng-Yee Chang, Yin-Ching Chuang, Balaji Veeraraghavan, Anucha Apisarnthanarak, Maria Fe R Tayzon, Lay Hoon Andrea Kwa, Cheng-Hsun Chiu, Zakuan Zainy Deris, Suraya Amir Husin, Hazimah Hashim, Anis Karuniawati, Altaf Ahmed, Tetsuya Matsumoto, Van Kinh Nguyen, Dinh Thi Thu Huong

**Affiliations:** 1 Tri-Service General Hospital/National Defense Medical Center, Taipei City, Taipei, Taiwan; 2 Chi Mei Medical Center, Tainan City, Tainan, Taiwan; 3 Christian Medical College, Vellore, Tamil Nadu, India; 4 Thammasat University Hospital, Pratumthani, Pathum Thani, Thailand; 5 Ateneo De Manila University, Pasig City, National Capital Region, Philippines; 6 Singapore General Hospital, Singapore, Not Applicable, Singapore; 7 Chang Gung Memorial Hospital, Kweishan, Taoyuan, Taiwan; 8 Universiti Sains Malaysia, Kota Bharu, Kelantan, Malaysia; 9 Ministry of Health, Malaysia, Kajang, Selangor, Malaysia; 10 Universitas Indonesia, Jakarta, Jakarta Raya, Indonesia; 11 Pakistan Kidney and Liver Institute, Lahore, Punjab, Pakistan; 12 International University of Health and Welfare, Narita-shi, Chiba, Japan; 13 Ha Noi medical University, Dong Da, Ha Noi, Vietnam; 14 National Hospital for Tropical Diseases, Ha Noi, Ha Noi, Vietnam

## Abstract

**Background:**

Most studies on hospital antimicrobial stewardship (AMS) status and practices are conducted in the west, and there is a lack of such data from Asian countries. The objective of this survey was to determine existing AMS practices and gaps, and challenges in implementing AMS programs in secondary and tertiary acute-care hospitals in 10 Asian countries.

**Methods:**

A 70-item questionnaire was disseminated to hospitals fulfilling inclusion criteria and responses were collected from 10 April 2020 to 9 April 2021. The survey, specific to the Asian hospital setting, enquired about hospital leadership support for AMS; AMS team membership and training; AMS interventions; AMS monitoring and reporting; hospital infrastructure; and education. These were subdivided into core and supplementary components, adapted from the Transatlantic Taskforce on Antimicrobial Resistance set of core and supplementary indicators for hospital AMS programs, and the US Centers for Disease Control and Prevention checklist for core elements of hospital AMS programs.

**Results:**

A total of 349 hospitals from Cambodia, India, Indonesia, Japan, Malaysia, Pakistan, Philippines, Taiwan, Thailand and Vietnam responded. Overall, only 47 hospitals fulfilled all 12 core components, and there were inter-country differences in terms of performance. The hospitals generally did well in terms of the AMS team (ie, comprising at least a physician leader responsible for AMS activities, a pharmacist, and infection control and microbiology personnel), and access to a timely and reliable microbiology service, with mean positive response rates (PRR) of ≥ 80% for these indicators (Figure 1). In the core components of AMS program interventions, and AMS monitoring and reporting, the lower mean PRR ( > 60%) revealed that Asia has wider gaps in these areas versus gold standards. Although many hospitals had formal hospital leadership statements to support AMS (mean PPR 85.6%), this was not always matched by allocated financial support for AMS activities (mean PPR 57.1%).

Figure 1

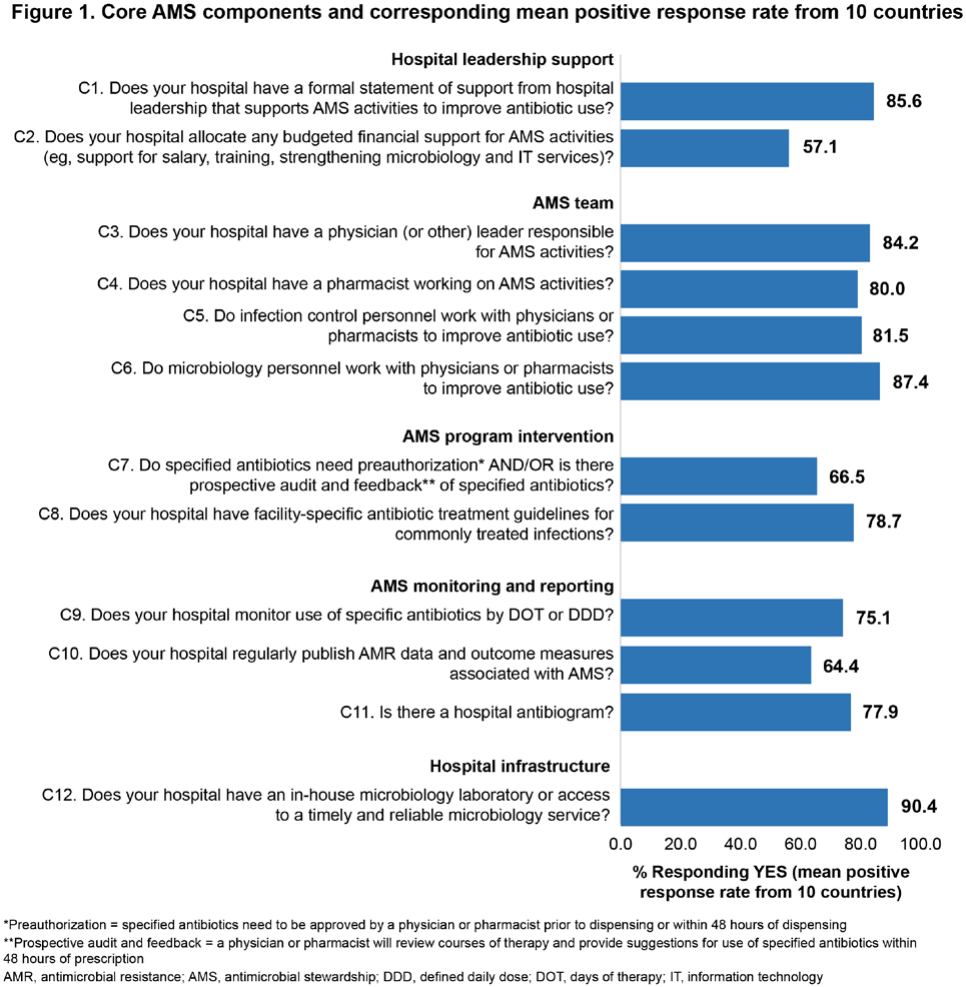

**Conclusion:**

For all core components of an AMS program, most Asian hospitals participating in this survey fell short of international gold standards. Inter-country differences in gaps highlight that country-specific solutions are needed to improve current standards in AMS.

**Disclosures:**

**Tetsuya Matsumoto, MD; PhD**, **MSD** (Speaker's Bureau)**Pfizer** (Speaker's Bureau)

